# The “New Code” Indorsed

**Published:** 1883-03

**Authors:** 


THE "NEW CODE" INDORSED.—By a vote of 105 to 99 the State Medical Society of New York has approved the new code of ethics by which allopathic physicians are allowed to consult with any legally qualified practitioner. The question of appr val led to a four-hours' debate, Dr. Squibb leading the opposition to approval, and Dr. D. B. St. John Roosa and Dr. C. R. Agnew leading the liberals. Dr. Roosa said, among other things: "It is assumed that, if we continue this code in force, we will immediately enter into brotherly relations with the homœopathists or eclectics. It is not so. We shall not ask them for assistance, but if any poor, uninstructed human being wants assistance we want the right to give it. We are not going to surrender to homœopathists. This is not a question of drugs; it is a question of ethics. The whole American educated public has been laughing at this restricted trade-union code."

The "new code" is causing quite a nice little family quarrel among our dear brethren. What now will the great American Medical Association do?

Dr. Austin Flint says: "If we refused to consult with homœopathists in the days when they honestly believed in Homœopathy, how can we consult with them now, when they are no longer honest?" Don't do it, Austin!

